# Disclosure, Privacy and Workplace Accommodation of Episodic Disabilities: Organizational Perspectives on Disability Communication-Support Processes to Sustain Employment

**DOI:** 10.1007/s10926-020-09901-2

**Published:** 2020-05-14

**Authors:** Monique A. M. Gignac, Julie Bowring, Arif Jetha, Dorcas E. Beaton, F. Curtis Breslin, Renee-Louise Franche, Emma Irvin, Joy C. Macdermid, William S. Shaw, Peter M. Smith, Aaron Thompson, Emile Tompa, Dwayne Van Eerd, Ron Saunders

**Affiliations:** 1 Institute for Work & Health, 481 University Avenue, Suite 800, Toronto, ON M5G 2E9 Canada; 2 Dalla Lana School of Public Health, University of Toronto, Toronto, ON Canada; 3 WorkSafeBC, Vancouver, BC Canada; 4 School of Physical Therapy, Western University, London, ON Canada; 5 University of Connecticut Health, Farmington, CT USA; 6 Workplace Safety and Insurance Board (WSIB), Toronto, ON Canada; 7 Faculty of Medicine, University of Toronto, Toronto, ON Canada

**Keywords:** Chronic disease, Episodic disability, Employment, Disclosure, Accommodation, Support, Communication, Mental health

## Abstract

*Purpose* Employers increasingly are asked to accommodate workers living with physical and mental health conditions that cause episodic disability, where periods of wellness are punctuated by intermittent and often unpredictable activity limitations (e.g., depression, anxiety, arthritis, colitis). Episodic disabilities may be challenging for workplaces which must comply with legislation protecting the privacy of health information while believing they would benefit from personal health details to meet a worker’s accommodation needs. This research aimed to understand organizational perspectives on disability communication-support processes. *Methods* Twenty-seven participants from diverse employment sectors and who had responsibilities for supporting workers living with episodic disabilities (e.g., supervisors, disability managers, union representatives, occupational health representatives, labour lawyers) were interviewed. Five participants also had lived experience of a physical or mental health episodic disability. Participants were recruited through organizational associations, community networks and advertising. Semi-structured interviews and qualitative content analysis framed data collection and analyses, and mapped communication-support processes. *Results* Seven themes underpinned communication-support process: (1) similarities and differences among physical and mental health episodic disabilities; (2) cultures of workplace support, including contrasting medical and biopsychosocial perspectives; (3) misgivings about others and their role in communication-support processes; (4) that subjective perceptions matter; (5) the inherent complexity of the response process; (6) challenges arising when a worker denies a disability; and (7) casting disability as a performance problem. *Conclusions* This study identifies a conceptual framework and areas where workplace disability support processes could be enhanced to improve inclusion and the sustainability of employment among workers living with episodic disabilities.

## Introduction

The number of individuals living with a disability is increasing due to an array of influences, including an aging population, the growing prevalence of chronic conditions like musculoskeletal and mental health disorders, cancer, and cardiovascular diseases, and a wide range of social and environmental factors that contribute to disability [1]. Although the employment rate of individuals with a disability is lower than the general population, improvements in health treatments and rehabilitation mean that many individuals are able to sustain or return to work, and they often expect to work longer than previous generations [1, 2].

### Episodic Disabilities and Employment

Of increasing relevance to workplaces are episodic disabilities. Episodic disabilities commonly arise from chronic conditions where there are times of comparative wellness punctuated by intermittent periods of more severe symptoms that can contribute to activity limitations [3]. They are frequently unpredictable even when health conditions are well managed by treatment. Moreover, many conditions resulting in episodic disability are described as invisible or hidden disabilities. That is, signs and symptoms of the condition may not be apparent to others until it is severe, or a person is undergoing an episode [3, 4]. Chronic diseases associated with episodic disability are often highly prevalent and include mental health disorders like depression and anxiety, rheumatic diseases like arthritis and lupus, Crohn’s and colitis, multiple sclerosis, migraine and epilepsy. Many musculoskeletal conditions like low back or neck pain and tendinopathies result in episodic disability as does chronic fatigue syndrome and other syndromes with unknown etiology. Improved treatments for previously life-threatening diseases like some types of cancer and HIV/AIDS have resulted in these also being cast as episodic disabilities.

Although diverse in their etiology, episodic disabilities may create common challenges for workplaces. This includes challenges that may arise when an organization must comply with legislation to protect an individual’s private health information and, at the same time, would benefit from obtaining personal information to meet a worker’s support needs. Organizations also may perceive challenges in balancing their responsibility to provide reasonable support and accommodations to workers living with episodic disabilities while maintaining health and safety obligations and productivity goals.

### Workplace Disclosure and Accommodation of Episodic Disabilities

Research on workplace disclosure and accommodation of episodic disabilities has focused almost exclusively on the perspective of workers, often those living with perceived “stigmatized identities” [5] related to mental illness and HIV/AIDS [6–12]. Studies highlight reasons given by individuals for their disclosure decisions; factors associated with disclosing; communication processes; and disclosure outcomes [4, 8, 12–24].

Motives for disclosing are wide-ranging and include needing workplace support, sharing to build trust, believing others have a right to know, and educating others to diminish stereotypes. Motives also can include disclosing to gain protection from legislation or being forced to communicate information because others notice a problem [10, 12, 25, 26]. Reasons for not disclosing are similarly varied and encompass concerns about the negative ramifications of communication, believing that private information is not others’ business, negative past experiences, wanting to avoid gossip, perceiving no need to communicate if a health condition does not impact the job, believing nothing can be done, self-stigma and wanting to “pass as normal” [10, 12, 25, 26].

Factors underlying workplace disclosure decisions have emphasized impression management, control of information, supervisor and co-worker relationships, and a worker’s expectations of the anticipated outcome of their communication decisions [8, 15, 19, 26–29]. Research also highlights that individuals vary in whether they partially or fully disclose or whether information is leaked or involuntarily disclosed [8, 12, 20, 26, 30]. The timing of disclosure (e.g., pre- versus post-hiring; career stage) [14, 31], crisis events [13, 30], and that disclosure is an ongoing, evolving process also have been underscored [12, 32]. Outcomes of communication decisions emphasize perceived stigma, prejudice or discrimination, affective responses (e.g., feeling hurt, angry), unwanted advice and negative social comparisons, but also include positive responses and the receipt of instrumental and emotional support [18–20, 26, 32, 33].

In Canada, laws aim to protect workers living with a disability, including legislation that guards personal health information and requires organizations to make reasonable accommodations for workers with a disability without access to diagnostic information [34, 35]. Workplaces are encouraged to focus on social and environmental barriers that can make employment difficult and not on medical diagnoses and symptoms. At the same time, health professional verification of an underlying condition that creates workplace activity limitations may be sought.

Currently, there are few studies examining workplace disclosure and support from an organizational standpoint [31, 36, 37]. This perspective is critical in addressing support gaps, understanding the interplay of key stakeholders, and in identifying new directions that can enable workers with episodic disabilities to better sustain employment or return to work. We aimed to better understand who is involved in the disability support process, how they interact and when; messages conveyed within organizations; and successes and challenges in implementing support, including when workers choose not to disclose an episodic disability to others. We used qualitative methods to gain insight into episodic disabilities from the perspective of individuals within a workplace who have responsibilities supporting workers living with episodic disabilities (e.g., supervisors, disability managers, union representatives). Because studies often examine workers with a single type of condition, we included organizational perspectives across a range of physical and mental health conditions causing episodic disability to identify common and dissimilar themes.

## Methods

### Participants

Purposive sampling identified supervisors, human resource (HR) professionals, disability managers (DMs), worker advocates (e.g., union representatives), occupational health professionals, health and safety representatives, and labour lawyers who had experience interacting with individuals living with episodic disabilities. In sampling, we sought women and men with different workplace roles, as well as who worked in diverse employment sectors across small, medium and large organizations. We sought perspectives from individuals who not only provided support to others living with episodic disabilities as part of their job responsibilities but who also reported that they had “lived experience” (i.e., they were someone who had a chronic physical or mental health condition that caused periods of disability at work). Potential participants were identified using a range of sources, including employer networks established at the Institute for Work & Health (IWH) (e.g., disability managers network, Ministry of Labour contacts); health charities serving individuals with episodic disabilities and their links to workplaces (MS Society of Canada, Arthritis Society, Realize Canada); the IWH newsletter and website; and various associations (e.g., Mental Health Commission of Canada). Most recruitment was via electronic information letters, but some posters were used at health charity conferences and events. Interested individuals contacted the study coordinator by email or telephone, were provided with additional information to assess their interest in the study and screened for study eligibility. To be eligible participants needed to speak English, be currently employed, and have workplace experience supporting workers with episodic disabilities. Recruitment continued until saturation of themes was reached.

### Procedure

We used qualitative content analysis and methods to guide the research. In-depth interviews lasting ~ 60 min were undertaken with participants in person or by telephone in 2017 and 2018. All interviews were conducted by MAMG and JB. Participants were informed that the study was part of a program of research aimed at gaining a better understanding of the complex workplace issues that arise when balancing communication, privacy and support needs of workers with episodic disabilities. Episodic disabilities were described to participants as chronic physical or mental health conditions where individuals often have periods of relatively good health punctuated by intermittent periods of poorer health resulting in limitations or disability at work. Examples of physical (e.g., arthritis, colitis, migraine) and mental health conditions (e.g., depression, anxiety) were provided to participants. Interviews were semi-structured. Questions asked participants: (1) to provide information about their role in the organization and what kind of experiences they had with episodic disabilities (including the type of episodic conditions with which they had experience, how they were addressed, successes and challenges); (2) their perceptions of awareness in their organization and in other organizations of episodic disabilities (including whether current policies and practices addressed support needs); (3) whether and how privacy legislation impacted support processes for workers living with episodic disabilities; (4) what they believed were the key issues related to a worker’s decision whether to share personal health information and support needs; (5) key people internal and external to the organization involved in the support process (including their roles, timing, examples of successes and challenges); (6) issues arising related to the intermittent nature of disability (including successes, challenges, timing of support, revisiting support over time); (7) any personal, health, work context or social and environmental factors that they believed were relevant in the support process (e.g., different preferences for privacy, past experiences, gender, age, type of job); (8) other issues perceived as relevant. All questions were probed for details and examples and interviews centred on the experiences of greatest relevance to each participant. Interviews were recorded, transcribed and entered in NVivo for analysis [38]. Informed consent was obtained from participants. Ethics approval was received from the University of Toronto Research Ethics Board (#33620).

### Analyses

Transcripts were analyzed using qualitative content analysis, a method for making inferences from verbal or text data through the development of a systematic coding process that identifies themes and patterns in the data [39, 40]. Because data with workplace participants are limited, we used conventional content analysis, which avoids preconceived categories. Initial coding began during interviewing with MAMG, a health and social psychologist, reading transcripts to achieve a sense of the data and to develop topic areas capturing key concepts and emerging themes. This formed the basis of an initial coding scheme and helped identify when saturation of themes was reached in interviews. The coding scheme was shared with JB and a research assistant. They independently coded a small number of transcripts. The coding scheme was revised to clarify and add new codes. All transcripts were double-coded, and the codes compared. Areas of divergence were discussed. Thematically similar codes were clustered into themes. To establish the credibility of the themes, they were shared with members of the research team and representatives of partner organizations involved in the grant, and as part of presentations given to individuals involved in disability support (e.g., disability managers, supervisors) and individuals who worked with an episodic disability (~ 20 individuals). As a result, some theme labels were clarified and the relationships among themes discussed. A tree diagram was developed to organize the communication-support process [40]. A final step in the thematic analysis was a directed content analysis where themes emerging from the research were compared to concepts discussed in previously published studies in this area.

## Results

Twenty-seven interviews were conducted with supervisors/managers (n = 4), DMs (n = 7), HR personnel (n = 5), worker advocates/union representatives (n = 5), labour lawyers representing either workers, a large union, or a large organization (n = 3), a medical director and occupational health nurse (n = 2), and a health and safety representative (n = 1) (see Table 1). Five participants spanning these occupations lived with a physical or mental health episodic disability. Most participants were women (n = 20) and all had extensive experience in their professions (range: 8 to 30 years). Participants worked in diverse sectors, six worked for small businesses and four were self-employed or owned their own business.

**Table 1:** Participant characteristics

	N	Mean (range)
Gender		
Female	20	
Male	7	
Years in profession		19.5 (8–30)
Occupation		
Disability manager	7	
Human resources personnel	5	
Manager/supervisor	4	
Worker advocate/union representative	5	
Labour lawyer	3	
Medical director	1	
Occupational health nurse	1	
Health and safety representative	1	
Participants living with an episodic disability	5	
Job sector		
Business, finance, professional services	4	
Education or government	6	
Healthcare	6	
Manufacturing, construction or utilities	4	
Non-profit	1	
Service or retail	1	
Served multiple sectors	5	
Organization size		
Small (< 100 employees)	6	
Medium or large (≥ 100 employees)	21	
Union present		
Yes	14	
No	7	
Sometimes (i.e., consultant may work with union)	6	
Self-employed/business owner	4	

**Fig. 1:**
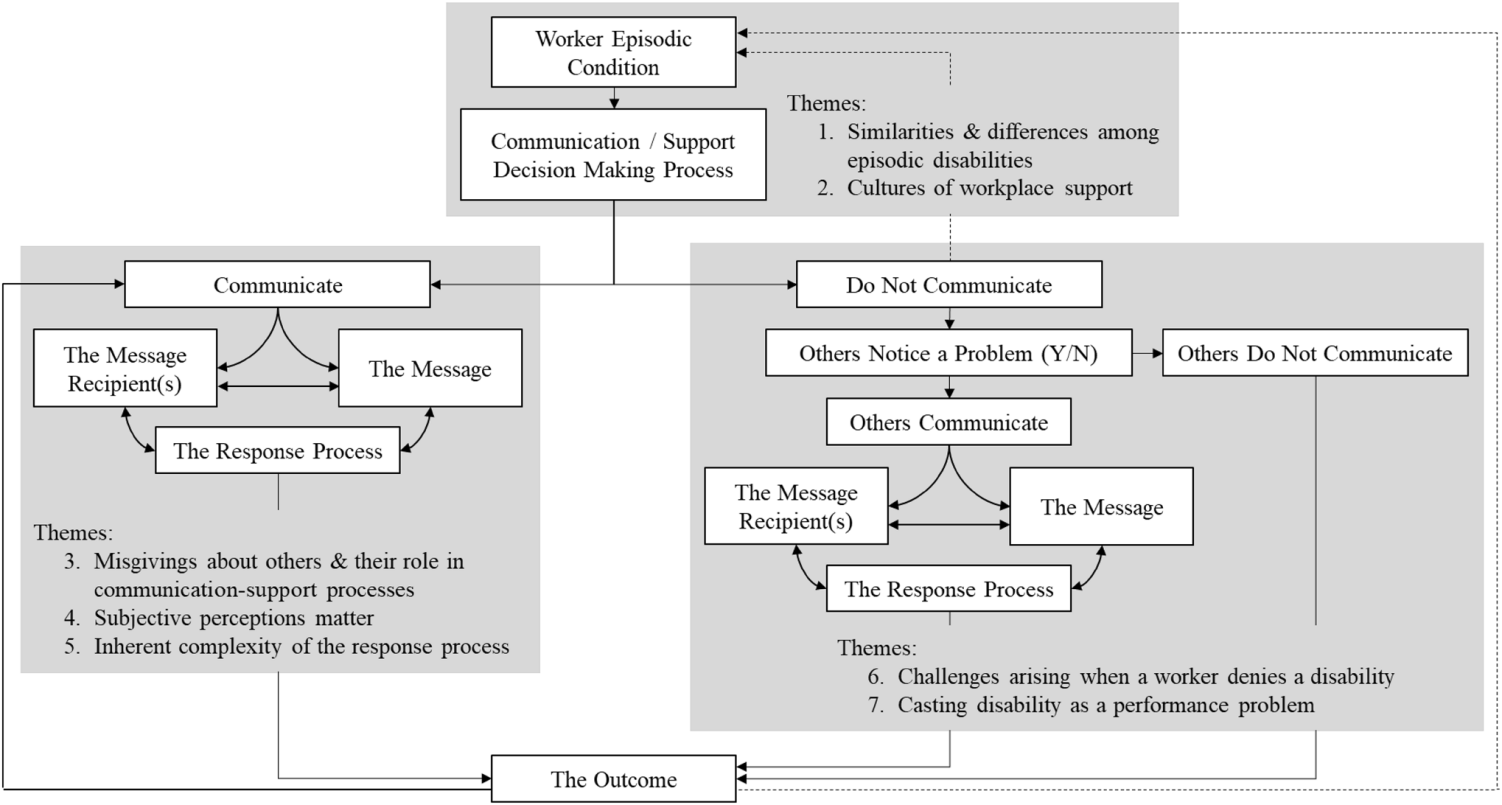
A framework of the communication-support process and themes

Figure 1 presents a framework that highlights the communication-support process. Three broad components of the framework are discussed: 1) themes relating to types of episodic disabilities and communication-support decision making processes; 2) themes emerging when a worker communicates information about their episodic disability at work; and 3) themes emerging when a worker does not communicate information about their episodic disability. Links among the boxes underscore the iterative and inter-connectedness of the communication-support process and are discussed along with themes. Unless noted, themes were similar across physical and mental health episodic conditions.

### Type of Episodic Disability and the Communication-Support Decision-Making Process

#### Theme 1: Similarities and Differences Among Episodic Disabilities

Respondents agreed that providing support to people with episodic disabilities was an important and growing issue at workplaces. Increased awareness of a range of conditions that caused episodes of disability, as well as changing workforce demographics were discussed. Many similarities among physical and mental health episodic disabilities were noted in their impact on job limitations, the work environment, absenteeism and presenteeism. Shared impacts suggested common policies could be implemented to provide support. “I’m always of the opinion, and even when people get into mental health cases versus physical cases—no, no, no—they’re all still disability cases. You can still apply the same procedure with your main goals” (Resp 13, DM, manufacturing). A notable exception was mental health conditions where individuals living with the condition sometimes lacked awareness of the onset of an episode. In these cases, interpersonal tensions sometimes arose in communication and support. One manager noted, “More commonly with a mental health condition, you’ve got subtler things: meltdowns, chronic lateness, inability to concentrate, disruptive behaviour, not fulfilling commitments, or not showing up for work regularly…We label them as complex cases, we try to be as good as we can. When somebody’s perception of their ability doesn’t match the reality, then we have to take those very delicately” (Resp 7, Manager, public sector). Another said, “It opens up a whole other level of activity if they’re paranoid and they think that the whole world is against them…. It’s a problem if they’re not aware. It’s really problematic.” (Resp 26, Manager & HR, public sector). Although participants noted these cases were rare and reflected more serious cases of mental illness, the time lapsed before mental health issues were recognized, including by a worker, could create long lasting, if not irreparable harm, to workplace relationships. Re-building positive work environments was complex and influenced other aspects of the communication-support process.

Communication-support decision-making discussions highlighted a growing awareness of physical and mental health episodic disabilities and gradual improvement in workplace attitudes; proactive organizational changes to facilitate communication and support; challenges in the processes; and the positive release experienced by some workers when they were able to share their needs and receive support. Yet study participants, especially those who lived with an episodic condition, also recognized why workers do not communicate personal information or wait until a crisis before communicating. Regardless of their health, many participants noted that workers with an episodic disability wanted others to maintain a positive impression of them, protect their job security and career development, varied in their preferences for privacy, and often had negative past experiences with stigma. One respondent living with an episodic disability noted, “There is a lot of stress that goes along with telling people because, first of all, they look at you like you’ve got two heads, and then they treat you like you’re very fragile… People become very concerned, which is—while it’s a nice feeling—it’s very limiting.” (Resp 27, small business owner with an episodic disability).

#### Theme 2: Cultures of Workplace Support

A key theme underpinning the decision-making process was the culture of support in an organization. Three dimensions were noted within this theme: (1) medical versus biopsychosocial models of support; (2) how best to promote fairness and transparency; and (3) and a return on investment (ROI) that focuses on readily measurable financial returns versus a value on investment (VOI) business model that includes intangible or less easily measured monetary benefits, such as improvements in morale, and organizational advantages related to worker experience and loyalty (see Table 2). There was considerable diversity in perceptions with few participants questioning the culture within their organizations and many participants perceiving that their communication-support processes were preferable to alternative processes. A few participants noted a lack of congruence in the perspectives adopted within their organization, making support of workers particularly challenging.

**Table 2:** Cultures of workplace support

Medical versus biopsychosocial approaches to episodic disability support	
“Because our third-party providers have that [diagnosis], in most cases, it’s a much smoother transition…. I find even return to work recommendations are more meaningful because they have the diagnosis. As you know, the most important thing is that people are properly diagnosed.” *(Resp 8, DM, utilities)* “Insurance companies want medical documentation and…disability plans expect you, that if [person X] is going to be off for more than four months, she better be seeing a specialist. In other words, she better show some initiative to improve…” *(Resp 21, union representative, healthcare)*	“We only gather medical information, or I get involved in about 25% of cases. Seventy five percent of cases do not involve a medical practitioner at all for six months—up till they go to long-term disability. We would sort of describe that as a continuous improvement thing where we’re trying to accommodate people as opposed to manage their diagnosis, which is a complete and utter waste of time…. You can’t explain everything by medicine… and you need to find some way to be fleet of foot and manage these because, if you don’t, they go sour very quickly.” *(Resp 9, medical director, business/finance)* “I think it’s the biggest single challenge and opportunity that we’ve got… A lot of people don’t even have a reliable healthcare provider so this whole thing of ‘get a sick note after five days, what are your limitations’—it’s really a fiction…. We have so little practical preparation for these health professionals to play the role they need to play.” *(Resp 7, worker advocate, public service)*
Promoting fairness and transparency	
“Supervisors aren’t supposed to just make a side deal with the workers…. Because then you’re sort of making side deals with everybody, but nothing is really documented. It’s super important that, if there’s a concern, that it go forward to either to the HR or health and safety individual.” *(Resp 13, DM, manufacturing)*	“It’s impossible, I think, to capture in any sort of policies or procedure the degree of nuance and individualization and the contextual analysis that you have to bring to bear on this sort of stuff. It isn’t a one-stop. There isn’t the one easy answer. There isn’t the one fix. It has to be really individually tailored for everybody.” *(Resp 3, labour lawyer representing workers)*
Return on Investment (ROI) versus Value on Investment (VOI)	
“Everyone is interested in ROI and we’re trying to talk to them about value on investment instead. The VOI is really important…. My feeling is, if you do the right thing, the numbers will follow. I think if more organizations looked at it that way, they would also be looking at protecting psychological health and taking care of their employees. I think there is a shift that is happening. I’m just not sure that we are there yet.” *(Resp 20, DM, consulting firm)*	

Medical models of support were common and emphasized the validation of workers’ health claims with ongoing physician documentation and medical treatment. This approach was frequently adopted in large organizations or unionized environments where there was experience with workplace injuries, a strong tradition of health and safety activity, and collective agreements outlining processes and procedures. However, some organizations had moved to alternate support policies, recognizing health as only one determinant of disability. They adopted a biopsychosocial approach, emphasizing person-job fit (i.e., the congruence between the competencies and needs of the worker and the requirements of a particular job) and the physical and social environment that can contribute to disability or enhance the person-job fit. Supporting their decision to move beyond a medical model, participants noted challenges their workers encountered in accessing health care in a timely fashion, especially specialist care for physical or mental health conditions; hesitancy among some employees in having a mental health condition identified through letters that came from a psychologist or psychiatrist; a perception in the workplace that physicians lack experience to provide workplace disability support; and the need to act quickly to support and manage work limitations arising from episodic disabilities (see Table 2).

Promoting fairness and transparency in support processes was an aspiration of all participants. Yet, participants diverged in how this should be achieved, especially in their attitudes toward a case-by-case approach to episodic disability support. Some organizations perceived a case-by-case approach as haphazard and arbitrary and believed standard and uniform practices were needed. Others believed a case-by-case approach was flexible and individually responsive. They felt that individual differences, diversity in job demands, differences in episodic disabilities, and changes in health over time, made a case-by-case approach the only tenable process. Fairness and transparency were achieved with policies that communicated to workers that their needs would be addressed in a collaborative process (see Table 2).

ROI versus VOI perspectives were discussed with most participants endorsing the need for a VOI culture of disability support. Yet, they perceived that the norm, including in their own organization, often focused on ROI with limited endorsement of the long-term value of supporting workers with episodic disabilities. A return-on-investment attitude sometimes extended to perceptions of the value of the work undertaken by HR personnel and DMs and the resources that should be allotted to them. For example, some HR participants and DMs reported that their efforts to build awareness, increase training, and provide accommodations for workers with episodic disabilities were seen by their senior management as expensive and time consuming and as not contributing to the bottom-line of the organization.

### Workers Communicate Information About Their Episodic Disability at Work

When workers disclosed information about their episodic disability, participants noted that their goal was typically to manage job-related activity limitations or absenteeism. The support process was frequently straightforward with few individuals involved, mostly a supervisor/manager or trusted colleague(s). An informal process within a work unit might be undertaken to discuss self-management strategies, changes to job activities, their organization or scheduling. Widely available workplace policies were typically implemented if available (e.g., flextime, work-at-home arrangements).

The involvement of other individuals like HR, DMs or union representatives often signalled a more complex or challenging process, such as when workers or supervisors wanted expert advice or advocates, where support needs were more substantial, or where worker-supervisor interactions had become problematic. This was true for both physical and mental health conditions. In rare cases, legal action was undertaken. The evolving response processes highlighted that organizational representatives were not passive recipients of information, but actively shaped the process. Messages and message recipients varied with not all individuals being privy to the same information. At times, supervisors and workers were not included in discussions and reported feeling side-lined and let down by the support process. Themes highlighted: (1) misgivings about others and their role in communication-support processes; (2) that subjective perceptions matter; and (3) the inherent complexity of the response process.

#### Theme 3: Misgivings About Others and Their Role in Communication-Support Processes

Participants acknowledged the important roles others played in supporting individuals with episodic disabilities. However, comments frequently included concerns about the skills, training, motivation or involvement of other groups (see Table 3). For example, front-line supervisors were key gatekeepers who first recognized difficulties experienced by workers with episodic disabilities. Yet, variability in their interpersonal skills, training and experience meant that other participants (e.g., HR professionals, disability managers, labour lawyers) were wary of a supervisor’s effectiveness in providing support. Co-workers could be extremely supportive but could feel burdened by support processes that demanded time and extra work for them. Health professionals were essential in the medical management of episodic conditions but were perceived by participants as lacking the training or motivation to adequately participate in workplace support. Unions were important advocates for workers but promoting “special treatment” for a worker with an episodic disability could engender perceived inequities and conflict with other workers. HR personnel and DMs had training in communication-support processes. However, they were sometimes included late in the support process when interactions had become problematic. Some participants noted that variable experience and high HR turnover resulted in a lack of consistency in the process, and that HR staff were sometimes perceived by employees as representing the interests of the organization and not the worker. The involvement of labour lawyers signalled an adversarial situation, including perceived workplace discrimination.

**Table 3:** When workers communicate about their episodic disability: Misgivings about others and their role in the communication-support process

Supervisors and managers	“They just don’t have any sort of broad basis of knowledge upon which to base things. So, they are often coloured by stereotypes or predispositions and unknown discriminatory attitudes that they might have, and not even be aware of it.”	*(Resp 2, labour lawyer, workers)*
	“I think we probably have the right policies and practices in place…But, I can tell you, I bet most managers are not familiar with them. Most managers don’t have the awareness to even identify or even think about it being something other than a performance issue.”	*(Resp 26, HR & manager, public services)*
Co-workers	“It is sometimes difficult to let that history go. There’s still crews that are resistant to having somebody come back because the relationship was severed and somewhat toxic.”	*(Resp 8, DM, utilities)*
	“I think where the frustration kind of boils over is…No one is actually being open so that their team and their colleagues understand that this is not going to be fixed. This is the way it’s going to be. And I think it’s easier for people if they have—the more information they have, the better it is.”	*(Resp 12, HR, healthcare)*
	“They’re perceived as getting favourable treatment and then all the other co-workers are having to pick up the slack.”	*(Resp 5, DM, healthcare)*
Health professionals	“The physician role is really to diagnose and treat and we need to stop asking them if the person can do their job…They are very intelligent people, they certainly have the ability, but they do not have the time to understand the workplace.”	*(Resp 17, DM, consulting firm)*
Union representatives	“In the unionized environments that I worked at previously, they tend to compare one person against another—‘why did you do this for this person and not something for this person?’”	*(Resp 1, HR, service sector)*
HR and disability managers	“I did find that the turnover in that group was quite high…Even mid-process…I was dealing with one person and then all of a sudden they had moved on…That continuity, just organizationally, was a challenge.”	*(Resp 15, manager, public sector)*
	“HR doesn’t normally interact with the employees—I know that sounds a bit odd. It’s HR to manager, manager to employee…. Their name is human resources, you would think that they have hands on, but no… they’re sort of one step removed.”	*(Resp 25, manager, healthcare)*

#### Theme 4: Subjective Perceptions Matter

The role of subjective perceptions in influencing the communication-support process was acknowledged by participants, many of whom advocated for better awareness of stereotypes, preconceptions, and biases, yet believed it was naïve to think they were entirely avoidable. This theme highlighted that, behind workplace policies and practices, there are individuals with a range of experiences and perceptions who are implementing the policies. The most common challenge discussed by participants was grappling with not knowing the health diagnosis underpinning an episodic disability (see Table 4). This was true for both physical and mental health conditions, but particularly the case for suspected mental health conditions where absenteeism, productivity and interpersonal issues could be labeled as problems with skills, motivation, and performance. Participants recognized it was human nature to want to understand more about another’s health, partly out of curiosity, but also because they believed it was easier to provide support and avoid making mistakes if they understood the problem. Yet, participants also recognized that confidentiality surpassed their desire for information, and they supported policies that protected privacy. They discussed efforts to discourage gossip in the workplace, as well as the challenges that ensued when they were charged with protecting privacy, but others were aware of a person’s health status. One participant commented on privacy conundrums she experienced when workers would come to her seeking information. She said, “In a small office—you know how it is—people actually have relationships and so confidentiality is out of the bag. We couldn’t have tried to pretend that [person X] wasn’t away on a mental health disability. The place is just too small, and everybody saw the symptoms themselves. What position does HR have to take?” (Resp 14, HR, professional services). Participants also recognized interpersonal differences in attitudes, beliefs and perceptions played a key role in shaping support processes, despite the goal to provide everyone with comparable support (see Table 4).

**Table 4:** Themes arising when workers communicate about their episodic disability: Subjective perceptions and the inherent complexity of disability support

Subjective perceptions matter: It’s human nature to wonder and people impact the process	
“People get curious. It becomes the puzzle of the week…They said that they have difficulty with focus, and they said that they have difficulty with this. I’m thinking it must be this. Oh, no, it must be this… What is it? What is it?”	*(Resp 02, labour lawyer, workers)*
“I can’t tell you how terrible it feels not to know what it is. Not because we’re nosy people, but from a support perspective.”	(Resp 20, DM, consultancy business owner)
“There’s some workers who just—they have a way about them, and they explain themselves, and you hear them. Then there’s other workers that are just more aggressive or demanding, and it doesn’t mean that their issue has less merit, but…people just sometimes start to shut those people down because they can’t see past…their personality, and [that] there’s a real issue here.”	*(Resp 10, worker advocate)*
“[It matters] whether you start from a position of, ‘I’m going to trust until I have reason not to trust’, or if you start from, ‘I’m not going to trust until you give me reason to trust.’”	*(Resp 12, HR, healthcare)*
The inherent complexity of the response process	
“The paradigm of an intermittent episodic illness is completely different because you don’t know when conditions are going to flare-up and for how long. It is virtually impossible then, to plan around it, unless you’ve got, essentially, extra resources in the workplace…but most employers now are running very, very lean.”	*(Resp 4, lawyer, employer)*
“The major thing you’ll hear from the operations side is they need to know when someone is going to be here doing their work…. They always want us to try and quantify when someone has a condition exactly how many days a week that means or exactly how many times a month or year it’s going to be affected. And that’s not possible.”	*(Resp 16, DM, education)*
[Are people supportive?] “Yes, for the most part. But you know, it depends on how long it goes. And I’ll be honest about that because everybody has a lot on their plate. Everybody wants to be supportive, but we have to make sure that’s not creating stress and anxiety for the people who are left behind at the office.”	*(Resp 18, HR, not-for-profit)*
“It’s fatigue, I think. Employers will [cite] those multiple efforts and the fact of unsuccessful efforts to slowly build a case for undue hardship.”	*(Resp 3, lawyer, union)*
“We have problems with…any invisible conditions. People say, well, there’s nothing wrong with them. Why do they need accommodation? Look at them. They look fine…The person is milking the system. They just want it easy.”	*(Resp 23, union representative, multiple sectors)*
“I think we all wait until it becomes a problem…People don’t want to admit that there is a weak link or a weakness because they’re afraid that their senior manager is going to say, ‘just get rid of them, just fire them’”	*(Resp 14, HR, professional services)*

#### Theme 5: The Inherent Complexity of the Response Process

Participants recognized the need to improve workplace support for individuals with physical and mental health episodic disabilities. Yet, they acknowledged significant challenges impeding the process (see Table 4). Examples were provided where challenges were surmounted, but participants also noted cases where there was a breakdown in the process. For example, the intermittent nature of an episodic disability meant that work units reported that planning efforts to meet ongoing work demands was problematic. An absence of resources to mitigate workplace disruptions exacerbated planning efforts and respondents noted their colleagues felt frustrated if solutions could not be found quickly or if plans needed re-visiting or changing. The invisibility of symptoms meant that others sometimes viewed workers requesting support as malingering, which heightened interpersonal tensions in the workplace and interfered with support processes. Participants also noted that some individuals were omitted from disability support planning to protect the employee’s privacy. This was challenging when it included supervisors who were expected to implement plans, often with the assistance of other workers in the unit. Supervisors reported they were not given sufficient input or provided with the resources they needed to do this adequately. Finally, requests for support were often as a result of a health or work crisis. Participants noted that workers were understandably reluctant to discuss their health before a workplace problem occurred. However, the absence of proactive discussions further exacerbated planning efforts to meet work demands, could heighten interpersonal tensions, and delay accommodation and support efforts. These issues crossed both mental health and physical conditions, were noted across job sectors, and by participants with different support roles.

### Workers do not Communicate Information About Their Episodic Disability at Work

Participants recognized that many individuals do not disclose episodic disabilities at work. Respondents respected workers’ decisions and did not encourage indiscriminate disclosure of personal information. Yet, the most complex and potentially stressful situations participants faced often revolved around instances where a worker did not disclose their disability and others noticed a problem. This resulted in a decision-making process where others had to decide whether they would approach the worker and communicate their concerns. Participants reported that, in their experience, factors important to a worker’s non-disclosure decision included the current relationship the worker had with others, a worker’s past experiences, their comfort levels with personal discussions, and the nature of the behaviour or work impact. When participants, especially supervisors, chose not to communicate with a worker who they suspected had a health condition, increased monitoring of the worker’s behaviour occurred with communication decisions being re-evaluated as needed. Participants noted that greater training for these situations would be beneficial. Two themes arose that were particularly troublesome in a workplace: (1) when a worker denied any workplace difficulties; and (2) when disability was cast as a performance problem.

#### Theme 6: Challenges Arising when a Worker Denied a Disability

Particularly stressful for those providing support were instances when discussions were initiated about workplace difficulties or problematic behaviours and a worker denied there was a problem. This was more likely to occur in cases of suspected mental health disability (see Table 5). A worker advocate living with a mental health condition noted, “Truly with mania you generally don’t know anything is wrong. It’s very hard to have insight when one is manic … In fact, I had some insight, but I really needed friends to say, ‘What’s going on?’” (Resp 19, worker advocate with a mental health episodic disability). Efforts to move forward in these instances were typically prolonged and difficult, and involved heightened interpersonal tensions. Participants reported variable success in these situations. Support failures were instances where repeated worker denials of a suspected disability became labeled as poor performance and resulted in job termination.

**Table 5:** Themes arising when workers do not communicate their episodic disability: Denying workplace difficulties and casting disability as a performance problem

Challenges arising when a worker denies a disability	
“This one individual was saying that people were talking about her… Staff would come in and do some work, and she would think that they were spying on her…. we talked to the physician, the psychologist about it, trying to get some information about accommodation—is she getting the right kind of treatment or does she need any treatment?… She thought she was fine. We don’t know if she was or not…. But really, she came very close to being fired.”	*(Resp 1, HR, service sector)*
“People themselves, they may not see it. It may be a slow progression…People don’t see it and then suddenly…they start missing deadlines, showing up late for work, looking dishevelled…. If a person doesn’t realize, they’re just thinking, ‘I’m having a bad day.”	*(Resp 17, DM, disability consulting firm)*
Casting disability as a performance problem	
“What happens with episodic conditions is that they have incidental absences and… if they pass that ten-day threshold, then a progressive discipline approach is taken with them and that’s not always the right approach to take for someone who just needs time off periodically to attend to their health”	*(Resp 05, DM, healthcare)*
“Too often where we find out as the representatives of the worker, it’s when they’ve come forward to get our assistance because they’re in a position of discipline. Because they’ve missed time from work, or their work performance is lacking, and they have not indicated that they have an issue and have tried to sort of hide it. Then suddenly it reaches a point where it’s now become discipline… That’s a really common situation for us.”	*(Resp 23, union representative, multiple sectors)*

#### Theme 7: Casting Disability as a Performance Problem

Several participants identified workplace programs, ostensibly designed to identify support needs early, as a double-edged sword in the disability communication-support process. Labelled attendance management or attendance support programs, they flagged employees with higher than usual absenteeism and mandated meetings with supervisors, HR staff or others. Employees had an opportunity to explain their absences, including sharing any health-related difficulties. Although participants noted that the program could identify workers with physical and mental health episodic disabilities earlier, they also believed that workers could feel “caught” and forced to disclose health issues they preferred to remain private; that workers were often ill-prepared with what to communicate and had little understanding of their rights and obligations; and that disability was now cast as a poor performance problem (see Table 5). Future conversations often continued to revolve around performance and could lead to an erosion of trust and good-will.

## Discussion

This study provides insight into organizational perspectives on episodic disability in the workplace and the communication-support processes used to assist affected workers to minimize work disability through accommodation and absentee management systems. Few studies have focused on insights from individuals within a workplace who are charged with providing support to workers with an episodic disability [31, 37]. The findings of this study, which involved a range of workplace roles, job sectors, and a variety of physical and mental health conditions, reaffirm prior research that has noted the complexity of the disclosure process. Yet, it goes beyond previous findings to develop a framework for the communication-support process and to consider similar and unique factors associated with different types of episodic disability; organizational culture; confidence and misgivings about others involved in disability support; subjective perceptions; and challenges arising from situations when the disability is not disclosed and impairments impacting work performance are addressed as a performance issue. The themes add greater specificity to concepts that are often vague in communication and support theories and point to gaps in the supports available to workers, supervisors, and co-workers regarding how to handle communication challenges. The framework and themes can be used to develop and test new research questions, act as a model of the communication-support process and be useful for health professionals and workplaces reviewing disability practices.

By focusing on physical and mental health episodic disabilities, we were able to better understand commonalities and unique areas of impact. Our findings suggest that many individuals providing support to workers believe it is feasible to implement practices with broad applicability across physical and mental health episodic disabilities by focusing on job needs. However, disrupted interpersonal dynamics was a more nebulous area of support. Interpersonal tensions were not viewed as an inevitable consequence of episodic disabilities, though it was recognized that they could sometimes arise and have an impact on the workplace. This was perceived as more common with mental health conditions, especially when a worker was not aware of changes in their behaviour. In general, this aspect of impact has not received attention in formal workplace policies or in previous studies. Greater awareness and workplace training for early identification and intervention may be useful for workers and workplace parties providing support, as well as additional research.

Culture is noted as relevant in communication theories but has been largely unexplored in workplace disability research [9, 12, 17]. This study highlighted three dimensions of workplace culture needing additional assessment and evaluation. The first was the extent to which organizations rely on medical models of disease in disability support and the implications for workplaces. Participants universally acknowledged the importance of health care professionals in treatment of health conditions. However, many believed that manifestations of a disability at work highlighted social and environmental issues that went beyond medicine. Similar findings have been found in research on work injury [41]. In cases where organizations relied on health professionals to verify the existence of an underlying condition, barriers to access created disruptive delays in organizational support. Moreover, verification by some specialists (e.g., psychiatrists, rheumatologists) “outed” a worker, creating concerns about privacy and stigma. Yet, many organizations were reluctant to abandon medical models, citing concerns about potential malingering among workers with conditions that were invisible and intermittent. The findings point to the importance of more explicit discussions of organizational culture and challenge workplaces to go beyond medical models in disability support.

Discussions about culture also reflected the difficulties organizations face in being consistent and transparent in implementing policies across diverse conditions and job demands, and the resources and value they place on sustaining the jobs of individuals with episodic disabilities. This is an evolving area, with organizations being concerned about escalating costs and wanting to demonstrate a measurable impact of disability support programs. Research is needed to address these concerns and demonstrate how programs and policies can be tailored to meet individual needs while being transparent and fair, and measure long-term outcomes related to work productivity and job sustainability.

Previous research shows that many workers desire to keep health information private [10, 12, 25, 26]. Participants in this study recognized and respected this choice, but their discussions indicated that many individuals typically play some role in disability support over time making consistency of accommodations and the maintenance of privacy difficult [31]. Of concern are the misgivings participants had of others related to their skills and abilities, even when motivation to help was positive. This was also highlighted in the subjective perceptions that individuals bring to disability support. These findings signal the need for cross-professional discussions regarding the provision of support; the unintended consequences of excluding others, including an erosion of trust when supervisors, co-workers, and even the worker with an episodic disability are left out of discussions; and the need for additional training and skills building across professions. Rather than ignore subjective perceptions, training should recognize the inherent curiosity individuals have in trying to understand the causes of a problem but help individuals within organizations find strategies to move beyond the desire for information that is not relevant to maximizing a worker’s potential at their job.

The intermittent nature and potential for health crises inherent in episodic disabilities made them different from more stable or permanent disabilities. This was clear in participants’ reports of the challenges and frustration experienced by their colleagues in workload planning and distribution of job tasks. To date, research has often focused on more stable, permanent disabilities. Greater attention to intermittent, hidden and unpredictable disabilities is needed.

Important themes in this research that have received little attention elsewhere relate to the processes that unfold when workers either deny job difficulties that others believe are occurring, or when existing policies inadvertently characterize an episodic disability as a performance problem that requires disciplinary action. An exception is a study by Williams-Whitt and Taras that examined Canadian labour arbitration cases and highlighted ways that disability was regularly cast as a performance, attendance or disciplinary issue [42]. Respondents in the current study recognized that there should not be an expectation that workers communicate private health information. However, they struggled to move forward in these situations, which were multifaceted and could become adversarial. The results point to several directions for future efforts, including finding ways for workers to receive support in advance of a work problem without having to share health information, and more emphasis on skills recognition in the communication-support process. Previous research with workers has emphasized control over information [9, 16] and the inadvertent “leaking” of health symptoms that sometimes occurs [12, 20, 26, 30]. This study highlights additional complexity. Workers may be particularly vulnerable when they are not aware of early changes in their behaviour. Workplaces may misconstrue an episodic disability as a performance problem and individuals providing support may not be well equipped to interpret or understand episodic health changes.

This research has several strengths and limitations. We included individuals with a range of organizational roles to provide diverse perspectives on disability support, and focused discussions on mental and physical health conditions that can result in episodic disabilities. This enhanced the richness of the data and yielded insight into communication-support processes and new themes for additional research. However, our study may not have captured all the processes and interplay among support providers and workers, or experiences in different job sectors and jurisdictions. Our methodology also made it difficult to examine some contextual factors that were not discussed by participants. For example, our participants generally did not comment upon gender, age, education or other factors that may be important to communication-support processes. Research using other methodologies and replicating our findings is needed.

Nevertheless, this study reveals the importance of understanding workplace communication-support processes from the perspective of those providing support to workers with episodic disabilities. The findings highlight issues arising when organizations aim to protect privacy and provide support, as well as challenges arising when workers choose to disclose or not disclose personal health information. It identifies areas of focus for future research, training, guidance materials, and policy review. It is critical to address support gaps, understand the interplay of key stakeholders in disability support processes, and identify ways to enable workers with episodic disabilities better sustain employment or return to work.

## References

[1] World Health Organization (2011). World report on disability.

[2] OECD (2010). Sickness, disability and work: breaking the barriers: a synthesis of findings across OCED countries.

[3] Prince MJ (2017). Persons with invisible disabilities and workplace accommodation: findings from a scoping literature review. J Vocat Rehabil.

[4] Gignac MA, Backman CL, Kaptein S, Lacaille D, Beaton DE, Hofstetter C (2012). Tension at the borders: perceptions of role overload, conflict, strain and facilitation in work, family and health roles among employed individuals with arthritis. Rheumatology (Oxford).

[5] Ragins BR (2008). Disclosure disconnects: antecedents and consequences of disclosing invisible stigmas across life domains. Acad Manag Rev.

[6] Vickers MH (1997). Life at work with “invisible” chronic illness (ICI): the “unseen”, unspoken, unrecognized dilemma of disclosure. J Workplace Learn.

[7] Ragins BR, Singh R, Cornwell JM (2007). Making the invisible visible: fear and disclosure of sexual orientation at work. J Appl Psychol.

[8] Jones KP, King EB (2014). Managing concealable stigmas at work: a review and multilevel model. J Manag.

[9] Greene K, Petronio S (2000). Disclosure of chronic illness varies by topic and target: the role of stigma and boundaries in willingness to disclose. Balancing the secrets of private disclosures.

[10] Goldberg S, Killeen M, O'Day B (2005). The disclosure conundrum: how people with psychiatric disabilities navigate employment. Psychol Public Policy Law.

[11] Emlet CA (2006). A comparison of HIV stigma and disclosure patterns between older and younger adults living with HIV/AIDS. AIDS Patient Care STDS.

[12] Chaudoir SR, Fisher JD (2010). The disclosure processes model: understanding disclosure decision making and postdisclosure outcomes among people living with a concealable stigmatized identity. Psychol Bull.

[13] Toth KE, Dewa CS (2014). Employee decision-making about disclosure of a mental disorder at work. J Occup Rehabil.

[14] Spirito Dalgin R, Bellini J (2008). Invisible disability disclosure in an employment interview: impact on employers' hiring decisions and views of employability. Rehabil Couns Bull.

[15] Robinson L, Kocum L, Loughlin C, Bryson L, Dimoff JK (2015). I wanted you to know: breast cancer survivors' control of workplace communication about cancer. J Occup Health Psychol.

[16] Petronio S, Durham WT, Baxter LA, Braithwaite DO (2008). Communication privacy management theory: significance for interpersonal communication. Engaging theories in interpersonal communication: multiple perspectives.

[17] Pachankis JE (2007). The psychological implications of concealing a stigma: a cognitive-affective-behavioral model. Psychol Bull.

[18] Oldfield M, MacEachen E, Kirsh B, MacNeill M (2016). Impromptu everyday disclosure dances: how women with fibromyalgia respond to disclosure risks at work. Disabil Rehabil.

[19] Munir F, Leka S, Griffiths A (2005). Dealing with self-management of chronic illness at work: predictors for self-disclosure. Soc Sci Med.

[20] Irvine A (2011). Something to declare? The disclosure of common mental health problems at work. Disabil Soc.

[21] Hadjistavropoulos T, Craig KD, Duck S, Cano A, Goubert L, Jackson PL (2011). A biopsychosocial formulation of pain communication. Psychol Bull.

[22] Gignac MAM, Cao X (2009). “Should I tell my employer and coworkers I have arthritis?” A longitudinal examination of self-disclosure in the work place. Arthritis Care Res (Hoboken).

[23] Garcia JA, Crocker J (2008). Reasons for disclosing depression matter: the consequences of having egosystem and ecosystem goals. Soc Sci Med.

[24] Dyck I, Jongbloed L (2000). Women with multiple sclerosis and employment issues: a focus on social and institutional environments. Can J Occup Ther.

[25] Smith SA, Brunner S (2017). To reveal or conceal: using communication privacy management theory to understand disclosures in the workplace. Manag Commun Q.

[26] Brohan E, Henderson C, Wheat K, Malcolm E, Clement S, Barley E (2012). Systematic review of beliefs, behaviours and influencing factors associated with disclosure of a mental health problem in the workplace. BMC Psychiatry.

[27] Westerman CYK, Currie-Mueller JL, Motto JS, Curti LC (2017). How supervisor relationships and protection rules affect employees’ attempts to manage health information at work. Health Commun.

[28] Roberts LM (2005). Changing faces: professional image construction in diverse organizational settings. Acad Manag Rev.

[29] Lassman F, Henderson RC, Dockery L, Clement S, Murray J, Bonnington O (2015). How does a decision aid help people decide whether to disclose a mental health problem to employers? Qualitative interview study. J Occup Rehabil.

[30] Hielscher E, Waghorn G (2015). Managing disclosure of personal information: an opportunity to enhance supported employment. Psychiatr Rehabil J.

[31] Jetha A, Yanar B, Lay AM, Mustard C (2019). Work disability management communication bottlenecks within large and complex public service organizations: a sociotechnical systems study. J Occup Rehabil..

[32] Gignac MA, Cao X (2009). "Should I tell my employer and coworkers I have arthritis?" A longitudinal examination of self-disclosure in the work place. Arthritis Rheum.

[33] McLaren RM, Steuber K (2013). Emotions, communicative responses, and relational consequences of boundary turbulence. J Soc Pers Relat.

[34] Office of the Privacy Commissioner of Canada. Privacy in the workplace. 2004. https://www.priv.gc.ca/en/privacy-topics/employers-and-employees/02_05_d_17/. Accessed 29 Oct 2019

[35] Ontario Human Rights Commission (OHRC). Human rights at work 2008—3rd Edition. Toronto, ON: The Ontario Human Rights Commission (OHRC); 2008. https://www.ohrc.on.ca/en/human-rights-work-2008-third-edition. Accessed 29 Oct 2019

[36] Norstedt M (2019). Work and invisible disabilities: practices, experiences and understandings of (non)disclosure. Scand J Disabil Res.

[37] Brouwers EPM, Joosen MCW, van Zelst C, Van Weeghel J (2019). To disclose or not to disclose: a multi-stakeholder focus group study on mental health sssues in the work environment. J Occup Rehabil..

[38] QSR International Pty Ltd (2016). NVivo qualitative data analysis software.

[39] Neuendorf KA (2002). The content analysis guidebook.

[40] Hsieh HF, Shannon SE (2005). Three approaches to qualitative content analysis. Qual Health Res.

[41] Kosny A, MacEachen E, Ferrier S, Chambers L (2011). The role of health care providers in long term and complicated workers' compensation claims. J Occup Rehabil.

[42] Williams-Whitt K, Taras D (2010). Disability and the performance paradox: Can social capital bridge the divide?. Br J Ind Relat.

